# The complete genome sequence of *Hafnia alvei* A23BA; a potential antibiotic-producing rhizobacterium

**DOI:** 10.1186/s13104-020-05418-2

**Published:** 2021-01-06

**Authors:** Opeyemi K. Awolope, Noelle H. O’Driscoll, Alberto Di Salvo, Andrew J. Lamb

**Affiliations:** 1 School of Pharmacy and Life Sciences, Robert Gordon University, Sir Ian Wood Building, Garthdee Road, Aberdeen, AB10 7GJ Scotland; 2 Graduate School, Robert Gordon University, The Ishbel Gordon Building, Garthdee Road, Aberdeen, AB10 7QE Scotland

**Keywords:** *Hafnia alvei*, *H. alvei*, Genome mining, Biosynthetic gene clusters, Antibiotics, Rhizobacteria, Bioremediation, Biocontrol, Plant growth-promoting rhizobacteria

## Abstract

**Objectives:**

The urgent need for novel antibiotics cannot be overemphasized. *Hafnia alvei* A23BA was isolated from plant rhizosphere as part of an effort to recover novel antibiotic-producing bacterial strains from soil samples. The genome of the isolate was sequenced to facilitate mining for potential antibiotic-encoding biosynthetic gene clusters and to gain insights into how these gene clusters could be activated.

**Data description:**

Here, we report the complete genome sequence of *H. alvei* A23BA obtained from the hybrid assembly of Illumina HiSeq and GridION reads. The genome, consisting of a circular chromosome and a circular plasmid, is 4.77 Mb in size with a GC content of 48.77%. The assembly is 99.5% complete with genomic features including 4,217 CDSs, 125 RNAs, and 30 pseudogenes. Thiopeptide, beta-lactone, siderophore, and homoserine lactone biosynthetic gene clusters were also identified. Other gene clusters of interest include those associated with bioremediation, biocontrol, and plant growth promotion- all of which are reported for *H. alvei* for the first time. This dataset serves to expedite the exploration of the biosynthetic and metabolic potentials of the species. Furthermore, being the first published genome sequence of a soil isolate, this dataset enriches the comparative genomics study of *H. alvei* strains.

## Objective

Bacterial secondary metabolites are invaluable sources of novel bioactive compounds. Many clinically useful antibiotics were derived from the secondary metabolites of soil dwelling bacteria [1]. However, only a small fraction of all known species have had their metabolites exploited in this way [2]. To this end, we sought to isolate novel antibiotic-producing bacterial strains from soil samples collected from the rhizosphere as antibiosis occurs naturally within it [3]. *H. alvei* A23BA was recovered as part of this effort.

*Hafnia alvei* is a Gram-negative, rod- shaped, facultatively anaerobic psychrotrophic bacterium. It is commonly isolated from clinical materials, gastrointestinal tract of animals, plant surfaces, soil, and water [4]. Some strains are commensals of the gastrointestinal tract while others are opportunistic pathogens implicated in both nosocomial and community-acquired infections [5, 6]. It is almost never associated with antibiotic production except for the antimicrobial activities reported for a strain isolated from the gut of honeybees [7]. Phylogenetic studies of this little-known species have shown its pan-genome to be open and dynamic with each strain possessing sets of unique genes [8]. Unique gene acquisition is mainly by horizontal gene transfer, and it reflects the adaptation of strains to their remarkably diverse natural habitats. Strains show considerable metabolic pathway diversity and varied biosynthetic potentials because of the open pan-genome making them good mining candidates for novel metabolites.

Consequently, the genome of *H. alvei* A23BA was sequenced to enable mining for potential antibiotic-encoding secondary metabolite biosynthetic gene clusters (smBGCs) that show little or no homology to known smBGCs. Furthermore, assembled genomes of *H. alvei* in public repositories are typically of clinical, human or food isolates, to the best of our knowledge, the complete genome sequence of *H. alvei* A23BA represents the first published complete genome sequence of a soil isolate.

### Data description

*H. alvei* A23BA was recovered from the rhizosphere of a garden plant in Aberdeen, Scotland (57.101 N 2.078 W). It was isolated using an ultra-minimal substrate medium (Data file 1) [9]. Upon isolation and strain purification, isolate was cultivated in nutrient broth (Oxoid, UK) at 37 °C for 24 h. Overnight culture was centrifuged and gDNA was extracted from pellets with the DNeasy® Ultraclean® Microbial Kit for DNA Isolation (Qiagen, UK). Isolate was preliminarily identified by 16S rRNA gene sequence comparison as *H. alvei* with 99% identity score.

Libraries were subsequently prepared from extracted gDNA by MicrobesNG (Birmingham, UK) for whole genome sequencing. For Illumina sequencing, libraries were prepared using the Nextera XT Library Prep Kit (Illumina, USA) and sequenced with the Illumina HiSeq system using a 250 bp paired end protocol. For GridION (Oxford nanopore) sequencing, libraries were prepared with Oxford nanopore SQK-RBK004 kit and/or SQK-LSK109 kit with Native Barcoding EXP-NBD104/114 (ONT, UK) using 400-500 ng HMW DNA. Sequencing was performed on a FLO-MIN106 (R.9.4 or R.9.4.1) flow cell in a GridION (ONT, UK).

Illumina sequencing run produced 4,973,530 short reads that were trimmed and paired using Trimmomatic [10] v0.30 with a sliding window quality cut-off of Q15. Ninety eight percent of reads were retained, and quality was assessed with FastQC [11] v0.11.8. Mean phred score across each base position was assessed with MultiQC [12] and found to be ≥ 28 (Data file 2) [13]. GridION sequencing run produced 18,642 reads with the mean read quality score of 10.5 (data file 3) [14] as assessed with NanoStat [15]. Paired short reads and long reads from GridION sequencing were assembled with Unicycler [16] v0.4.8.0. Assembly quality was assessed with QUAST [17] v5.0.2- two contigs (one chromosome and one plasmid) were identified with a total length of 4,772,047 bp, N50 value of 4,687,005 bp and #N’s per 100 kbp value of 0 (data file 4) [18]. Assembly completeness was assessed with BUSCO [19] v3.0.2 and found to be 99.5% (data file 5) [20]. Identity was confirmed as *H. alvei* by ANI analysis using the FastANI tool [21], with the ANI value of 97.8167. Gene and functional annotations were performed with PGAP [22] v4.11 and RASTtk [23]; pathways analyses were performed using the KEGG database [24] Rel 93.0 and the eggNOG mapper [25] vs 2.0.0. smBGCs were identified with antiSMASH [26] v5.0. Genome map was drawn with CGView [27] and presented in data file 6 [28].

In summary, the complete genome sequence of *H. alvei* A23BA is 4,772,047 bp in size with the overall GC content of 48.77% and sequencing coverage of 256.0 x. It comprises of one circular chromosome (4,687,005 bp; GC content 48.8%) and one circular plasmid (85,042 bp; GC content 47.2%). Genomic features include 4,217 CDSs, 25 rRNA, 92 tRNA, 8 ncRNA, 30 pseudogenes and 2 CRISPRs. Thiopeptide, beta-lactone (both showing little or no homology to known smBGCs) and siderophore smBGCs were identified (data file 7) [29]. Thiopeptides and beta-lactones are known for their antibiotic and/or anticancer activities [30, 31], while siderophores are used clinically as “Trojan horse” to deliver antibiotics to antibiotic resistant bacteria [32]. Gene clusters commonly associated with bioremediation, biocontrol, environmental adaptation, and plant growth promotion were also identified (data file 8) [33]. Please see Table 1 for links to data files 1–8.

**Table 1:** Overview of data files/data sets

Label	Name of data file/data set	File types (file extension)	Data repository and identifier (DOI or accession number)
Data file 1	Composition of ultra-minimal substrate growth medium	Portable Document Format file (.pdf)	10.6084/m9.figshare.12781193.v [9]
Data file 2	Quality distribution of Illumina reads	Joint Photographic Experts Group file (.jpg)	10.6084/m9.figshare.12643961.v1 [13]
Data file 3	Basic quality statistics of GridION sequencing data	Portable Document Format file (.pdf)	10.6084/m9.figshare.12781280.v1 [14]
Data file 4	Quast report	Portable Document Format file (.pdf)	10.6084/m9.figshare.12781289.v1 [18]
Data file 5	Short BUSCO summary	Joint Photographic Experts Group file (.jpg)	10.6084/m9.figshare.12643937.v1 [20]
Data file 6	Circular representation of *H. alvei* A23BA genome	Joint Photographic Experts Group file (.jpg)	10.6084/m9.figshare.12643949.v1 [28]
Data file 7	Predicted smBGCs of interest in *H. alvei* A23BA genome	Portable Document Format file (.pdf)	10.6084/m9.figshare.12781274.v1 [29]
Data file 8	Other gene clusters of interest in *H. alvei* A23BA genome	Portable Document Format file (.pdf)	10.6084/m9.figshare.13309049.v1 [33]
Data set 1	Illumina and GridION sequencing reads	Fastq file (.fastq.gz)	https://identifiers.org/ncbi/insdc.sra:SRP251948 [35]
Data set 2	Genome assembly of *H. alvei* A23BA	Fasta file (.fna)	https://identifiers.org/ncbi/insdc.gca:GCF_011617105.1 [36]

Given the quality control measures applied and results of analyses undertaken, we believe *Hafnia alvei strain A23BA chromosome, complete genome* [34] represents a high-quality dataset that would expedite the exploration of the biosynthetic and metabolic potentials of *H. alvei* A23BA and would also enrich the comparative genomics study of *H. alvei* strains.

## Limitations

This dataset was generated from a hybrid assembly to ensure accuracy and completeness. Furthermore, the hybrid assembler (Unicycler) autocorrects read errors and polishes final assemblies several times to ensure accuracy. Annotations and metabolic pathway analyses were carried out with robust and validated bioinformatics tools, and smBGCs were identified with the most comprehensive genome mining tool to date. Therefore, the authors are currently unaware of any limitations of the data.

## Data Availability

Data files 1–8 described in this Data note can be freely and openly accessed on Figshare (https://figshare.com/) [9, 13, 14, 18, 20, 28, 29, 33]. Data sets 1 and 2 can be freely and openly accessed on the NCBI database. Illumina and GridION reads generated have been deposited in the Sequence Read Archive under accession number SRP251948 (Data set 1) [35]. The genome assembly of *H. alvei* A23BA has been deposited in GenBank under accession number GCF_011617105.1 (Dataset 2) [36]. The BioProject accession number for the entire project is PRJNA610978. See Table 1 and references for details and links to the data.

## Abbreviations

GC: Guanine-Cytosine
CDSs: Coding sequences
RNA: Ribonucleic acid
rRNA: Ribosomal ribonucleic acid
tRNA: Transfer ribonucleic acid
ncRNA: Non-coding ribonucleic acid
CRISPRs: Clustered regularly interspaced short palindromic repeats
DNA: Deoxyribonucleic acid
gDNA: Genomic deoxyribonucleic acid
smBGCs: Secondary metabolite biosynthetic gene clusters
ONT: Oxford Nanopore technology
HMW: High molecular weight
#N’s: Average number of uncalled bases
ANI: Average nucleotide identity
